# Perceptual Sensitivity to Tactile Stimuli Is Enhanced When One Is Aware That Tactile Stimulus Intensity Is Self-Controlled

**DOI:** 10.3390/brainsci15030231

**Published:** 2025-02-22

**Authors:** Hitoshi Oda, Shiho Fukuda, Hiroshi Kunimura, Taku Kawasaki, Han Gao, Moritaka Futamura, Koichi Hiraoka

**Affiliations:** 1 School of Rehabilitation Science, Graduate School of Osaka Metropolitan University, Osaka 583-8555, Japan; hito_pt23@yahoo.co.jp (H.O.); vqnw01142@ares.eonet.ne.jp (S.F.); hiroshihayabusa821@yahoo.co.jp (H.K.); t.kawasaki@hakuho.ac.jp (T.K.); gh1823020@outlook.com (H.G.); 2 School of Medicine, Osaka Metropolitan University, Osaka 583-8555, Japan; sr22116e@st.omu.ac.jp

**Keywords:** tactile perception, efference copy, time prediction, self-triggered touch, electrodermal activity

## Abstract

Background and objectives: This study aimed to investigate whether perceptual sensitivity to tactile stimulus is affected by awareness of who controls the stimulus intensity. Methods: Thirteen healthy participants took part in this study. A participant held a dial and an experimenter held the other dial. One dial was to control the intensity of the tactile stimulus while the other (dummy dial) was inactive. The intensity of the tactile stimulus to the participant’s index finger providing each 1 s was increased by the participant or by someone else with or without the participants viewing a dial controlling the stimulus intensity. Results and conclusions: The stimulus intensity at the perceptual threshold, when controlled by the participant, was significantly lower compared to when controlled by someone else, regardless of visual availability. This indicates that awareness of the self-control of the tactile stimulus intensity enhances tactile sensitivity. The electrodermal level immediately preceding the stimulus at the perceptual threshold was significantly lower when the participant controlled the stimulus intensity compared to when it was controlled by someone else, with eyes closed. The electrodermal levels immediately before the perceptual threshold stimulus, when triggered by another person, were significantly higher with the eyes closed. These electrodermal findings suggest that cognitive stress is greater when the timing of the initial tactile perception is difficult to predict.

## 1. Introduction

The tactile perceptual process varies depending on whether the tactile stimulus is triggered by oneself or by someone else. For example, cutaneous reflex observed during gait was attenuated when the stimulus was self-induced [1]. Furthermore, the tactile perception was reduced when the tickling was self-induced [2,3,4]. In those two studies, participants remained at rest when tickled by someone else, but moved the hand when tickling themselves. Thus, the presence and absence of the motor command is the only difference between two conditions in these studies. Perceptual sensitivity to the tactile stimulus was suppressed during various movements, such as reaching, grasping, or elbow or finger movement, a phenomenon referred to as tactile gating [5,6,7,8,9,10,11,12,13,14]. Accordingly, the attenuation of the perception during self-induced tickle may be mediated by tactile gating.

Tactile sensitivity was attenuated when touch was triggered by active movement, but remained unchanged when the touch was triggered by passive movement or by someone else [15,16,17]. Efference copy of the motor command is used by the internal model to predict the perceptual sensation caused by the motor output [18,19,20,21,22]. This efference copy is likely generated when the tactile stimulus is self-induced because of the movement to trigger the stimulus. Thus, those previous findings suggest that tactile sensitivity is attenuated when the stimulus is predicted through the efference copy of the motor command [2,17,23,24,25,26]. Based on this view, if the tactile stimulus is equally predictable in both self-induced and externally triggered conditions due to same efference copy production, tactile sensitivity should not differ based on whether the stimulus is self-triggered or not (Hypothesis 1).

Tactile sensitivity increased when the timing of the stimulus could be predicted [27]. When a person tapped the left finger with the right finger, tactile sensitivity of the left finger was attenuated [16]. However, this attenuation decreased when the timing of the finger tap was either delayed or occurred earlier than expected. Similarly, tactile attenuation was reduced when the self-triggered tactile stimulus was delayed [15]. One interpretation of these previous findings is that the attenuation of the tactile sensitivity depends on the difficulty of predicting the stimulus timing. Accordingly, we hypothesized that the difference in the perceptual sensitivity between the self-triggered and externally triggered tactile stimuli is related to the difficulty of predicting the stimulus timing (Hypothesis 2). When the intensity of the tactile stimulus gradually increases from subthreshold to suprathreshold, individuals can predict the timing of their initial perception of the stimuli by visually monitoring and actively controlling the increase. Accordingly, if Hypothesis 2 is true, tactile sensitivity should differ between conditions where participants actively increase and visually monitors the stimulus intensity, and conditions where someone else controls the stimulus intensity without visual monitoring.

Variation of electrodermal activity reflects changes in sweat gland activity levels [28]. Short-term stressors, such as cold pressure, performing a Stroop task, or experiencing fear increase electrodermal activity levels (EDLs) [29,30,31,32]. Self-induced postural perturbation leads to an earlier electrodermal response compared to externally induced perturbation [33]. However, in this study, the self-generated trigger was initiated by the participant, while the externally generated trigger was initiated by another person. Therefore, efference copy of the motor command was present in the self-generated condition but absent in the externally generated condition. Accordingly, this difference may reflect the prediction of the stimulus based on efference copy of the motor command. In the present study, to test whether the effect of the self-generated tactile stimulus on sympathetic activity is due to the prediction of the tactile stimulus without the influence of efference copy (Hypothesis 3), EDL immediately before the stimulus whose intensity was at the perceptual threshold was compared between self-controlled and externally controlled stimulus intensities, while the participant generated efference copy of the motor command in both cases.

In the present study, we investigated whether perceptual sensitivity to the tactile stimulus is affected by awareness of who controls its intensity. Three hypotheses are tested in the present study. If self-controlled tactile stimulus and one controlled by someone else are equally predictable due to the same efference copy production, tactile sensitivity should not depend on whether the stimulus is self-triggered or not (Hypothesis 1). The difference in perceptual sensitivity between self-triggered and externally triggered tactile stimuli is related to the difficulty of predicting the stimulus timing (Hypothesis 2). The effect of the self-generated tactile stimulus on sympathetic activity is due to the prediction of the tactile stimulus without the influence of efference copy (Hypothesis 3). This investigation allows us to understand whether the tactile perceptual process is related to awareness of self-control of tactile stimulus intensity.

## 2. Materials and Methods

### 2.1. Participants

Thirteen healthy humans (eleven males and two females) aged 30.8 ± 10.5 years (20–48 years) participated in this study. The participants’ age was limited to between 18 and 50 years. We recruited 13 participants based on a previous study, which found a significant difference in perceptual threshold between conditions on the same number of participants [34]. The participants had no history of neurological or orthopedic disease. Twelve participants were right-handed and one was left-handed, according to the Edinburgh Handedness Inventory score [35]. Written informed consent was obtained from all participants. The experiment was conducted according to the Declaration of Helsinki and was approved by the ethics committee of Osaka Metropolitan University (Approval number: 2023-115, 1 August 2023).

### 2.2. Apparatus

A participant took a sitting position in front of an actual dial controlling the intensity of the tactile stimuli and a dummy dial that did not control it. An earmuff was placed over their ears. Ring electrodes (SL-100-1, Unique Medical, Tokyo, Japan) were placed over the index finger of the right hand. An anode of the electrodes was placed at the proximal phalanx and a cathode was placed at the middle phalanx. The distance between the electrodes was 1.5 cm. Electrical current to those electrodes was supplied by an isolator (SS-104J, Nihon Kohden, Tokyo, Japan) attached to an electrical stimulator (SEN-8203, Nihon Kohden, Tokyo, Japan). The duration of each electrical stimulus was 500 μs. Electrodes measuring the electrodermal resistance were placed over the tips of the right middle and ring fingers (T.K.K.2701, Takei Kiki, Tokyo, Japan). Electrodermal resistance was recorded during the test trials. The analog signals of electrodermal resistance were digitized using an A/D converter (PowerLab/8sp, ADInstruments, Colorado Springs, CO, USA) at a sampling rate of 10 kHz.

### 2.3. Baseline Stimulus Intensity at Perceptual Threshold

The stimulus intensity at the perceptual threshold, defined as the minimum stimulus intensity at which the participants could perceive the stimulus, was determined using the method of adjustment [36]. Firstly, an experimenter held an actual dial controlling the stimulus intensity, while the stimuli were provided each 1 s and increased the stimulus intensity until the participant perceived the stimuli. Secondly, the experimenter slowly decreased the intensity from this above-threshold level until the participant did not perceive the stimuli. Finally, the experimenter slowly increased the stimulus intensity again from this below threshold level until the participant perceived the stimuli. In all tests, participants responded “yes” as soon as they detected the stimulus. The stimulus intensity at the moment at which the participant first perceived the stimulus in this final trial was defined as the baseline stimulus intensity at the perceptual threshold.

### 2.4. Practice Trial

Before conducting the test trials, a practice trial was conducted. The electrical stimulus was provided each 1 s in this trial. The participant turned the actual dial controlling the stimulus intensity with the left hand so that the stimulus intensity increased from the intensity 4.7 V below the baseline stimulus intensity at the perceptual threshold until they perceived the stimulus. They kept the eyes open and watched the dial as they turned it. They memorized the speed at which the dial was turned during the practice trial so that they could replicate the same motion during the test trials.

### 2.5. Test Trials

**Figure 1:**
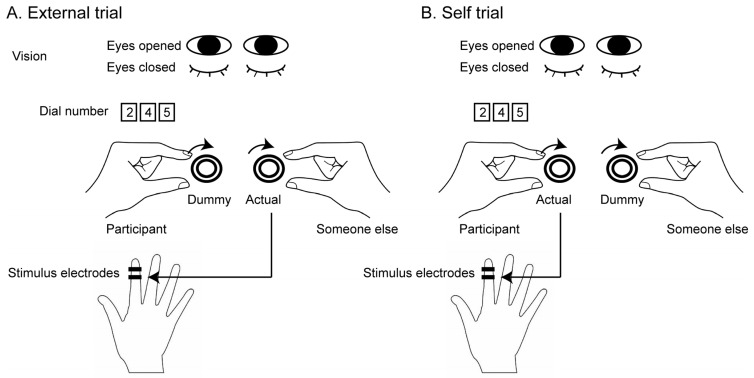
Experimental setup. (**A**) In the external trials, a participant turns a dummy dial but someone else turns an actual dial. (**B**) In the self trials, the participant turns the actual dial but someone else turns the dummy dial. The actual dial controls the stimulus intensity, but the dummy dial does not. They turn the dial to increase the stimulus intensity until the participant perceives the stimu-lus. In each trial, the eyes are closed or open. When the eyes are open, the participant views the number of the dial that is turned by them.

The test trials are shown in Figure 1. In the external trials, someone other than the participant held an actual dial controlling the intensity of the stimulus with the right hand, but a participant held a dummy dial where turning it with the left hand did not change the stimulus intensity (Figure 1A). Before conducting this trial, an experimenter verbally instructed the participant that the dial held by someone other than the participant was one controlling the stimulus intensity, but the dial held by the participant was a dummy. In the self-trials, the participant held an actual dial with the left hand but the other held a dummy dial with the right hand (Figure 1B). Before conducting this trial, the experimenter verbally instructed that the dial held by the participant was one controlling the stimulus intensity, but the dial held by someone other than the participant was the dummy. To confirm that the participant understood the instruction, they repeated what the experimenter instructed. Because of this procedure, the participant was aware of the person who controlled the stimulus intensity in each trial. Each trial involved one of two vision conditions: the eyes-open condition, where the participant kept their eyes open, and the eyes-closed condition, where the participant kept their eyes closed. The participant was instructed whether to keep their eyes open or closed before each trial. In the trial in which the participant kept the eyes open, they gazed at the number of the dial turned by them.

Each trial started when the fluctuation of EDL was minimal. The participant and someone other than the participant turned the dial, and the participant verbally responded “yes” when they finally detected the stimulus. At this point, the participant and someone other than the participant stopped turning the dial, and the experimenter recorded the number indicating the stimulus intensity in each dial. Four types of trials (self-trials with the eyes open, external trials with the eyes open, self-trials with the eyes closed, and external trials with the eyes closed) were conducted. Each type of trial consisted of 10 trials in an experiment. Those forty trials were randomly ordered.

### 2.6. Data Analysis

**Figure 2:**
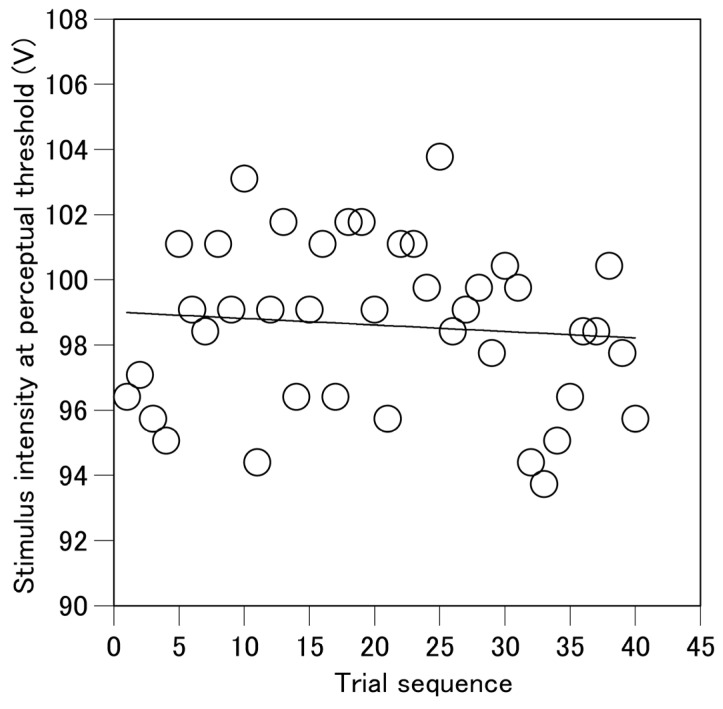
An example of the time series of the stimulus intensity at the perceptual threshold expressed as the voltage of the stimulus and a regression line of this intensity as the function of the trial series.

To determine the stimulus intensity at the perceptual threshold without influence of drift in the intensity across trials, an original data analysis was conducted. A regression line of the stimulus intensity at the perceptual threshold as the function of the sequence of the trials was estimated in each participant (Figure 2). This regression line represented the baseline level and trend of the change in the stimulus intensity at the perceptual threshold across the trials. Then, the residual for each stimulus intensity from this regression line was calculated. This residual represented the deviation of the stimulus intensity at the perceptual threshold from the baseline level without the influence of drift in the stimulus intensity at the perceptual threshold across the trials. Thus, the positive value of the residual means the increase in the stimulus intensity at the perceptual threshold and vice versa. Each residual was divided by the standard deviation of the residuals across the trials to calculate the z-score. The z-score indicated the stimulus intensity at the perceptual threshold as a magnification of the variability of the intensity across the trials. A positive z-score indicated an increase in the stimulus intensity at the perceptual threshold, and a negative z-score indicated a decrease in this value relative to the neutral stimulus intensity at the perceptual threshold across the trials. Pre-stimulus EDL (average EDL in the time window between 0 and 100 ms before the stimulus) in the trial in which the stimulus intensity was at the perceptual threshold was calculated.

### 2.7. Statistical Analysis

Repeated measures two-way ANOVA for the main effect of the controller (the participant or someone other than the participant, two levels) and vision (eyes open or eyes closed, two levels) was conducted. The result of Greenhouse–Geisser’s correction was reported whenever Mauchly’s test of sphericity was significant. The paired *t*-test with Bonferroni adjustment of alpha, comparing the mean number of the dummy dial and that of the actual dial at the perceptual threshold was conducted. The effect size of the main effect and interaction between the main effects in ANOVA was indicated by partial eta squared (η^2^p) calculated as the sum of squares of the effect in relation to the sum of squares of the effect and the sum of squares of the error associated with the effect [37]. The interpretation of Cohen’s *f* was, for instance, *f* = 0.1, small effect; *f* = 0.25, medium effect; and *f* = 0.40, large effect [38]. These *f* values correspond to η^2^p values of 0.0099, 0.0588, and 0.1379, respectively [37,39]. The alpha level was 0.05. Excel-Toukei 2016 ver. 3.21 (Social Survey Research Information, Tokyo, Japan) was used for statistical analysis. The data in the results are expressed as the mean and standard error of mean.

## 3. Results

### 3.1. Dial Number at Perceptual Threshold

**Figure 3:**
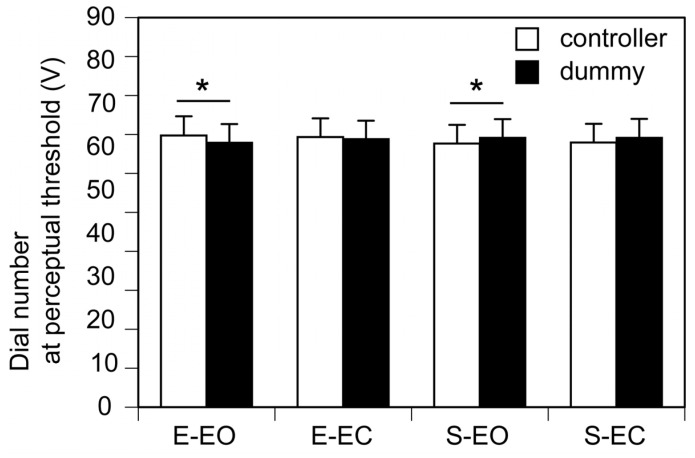
The dial number at the perceptual threshold. The dial number is calibrated as the actual voltage output. Bars represent the mean and error bars represent the standard error of mean. Each asterisk indicates a significant difference in the intensity between the controllers (*p* < 0.05). EO, the eyes open; EC, the eyes closed; S, self-trials; E; external trials.

The dial number at the perceptual threshold, calibrated as the actual voltage output, is shown in Figure 3. The number of the actual dial at the perceptual threshold was significantly greater than that of the dummy dial when the participants turned the dummy dial with their eyes open (*p* < 0.05). The number of the dummy dial at the perceptual threshold was significantly greater than that of the actual dial when the participants turned the actual dial with their eyes open (*p* = 0.046). Taken together, the number of the dial turned by the participant at the perceptual threshold was significantly lower when the participant’s eyes were open. In contrast, there was no significant difference between the number of the dummy dial and that of the actual dial at the perceptual threshold when the participant kept the eyes closed either when the participant turned the dummy (*p* = 1.947) or actual dial (*p* = 0.195).

### 3.2. Stimulus Intensity at Perceptual Threshold

**Figure 4:**
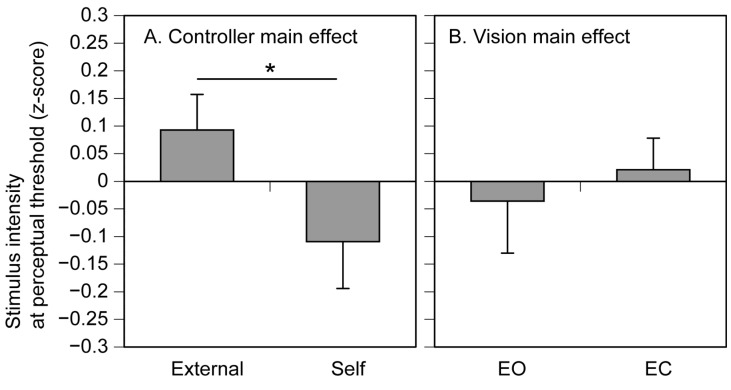
The stimulus intensity at the perceptual threshold expressed as z-score of residuals. Bars represent the mean and error bars represent the standard error of mean. An asterisk indicates a significant main effect (*p* < 0.05). EO, the eyes open; EC, the eyes closed.

The stimulus intensity at the perceptual threshold is shown in Figure 4. There was no significant interaction between the main effect of controller and that of vision [F (1, 12) = 3.078, *p* = 0.105, η^2^p = 0.204]. There was a significant main effect of the controller (F (1, 12) = 5.206, *p* = 0.042, η^2^p = 0.303); the stimulus intensity in the self-trials was significantly lower than that in the external trials. There was no significant main effect of vision (F (1, 12) = 0.122, *p* = 0.733, η^2^p = 0.010).

### 3.3. Pre-Stimulus EDL at Perceptual Threshold

**Figure 5:**
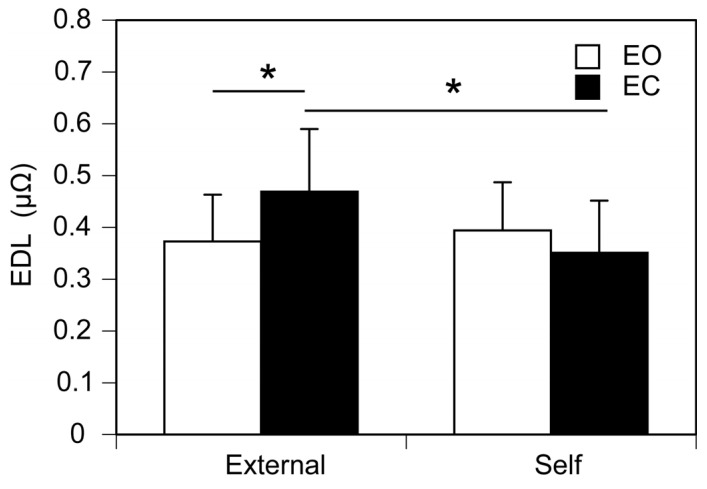
Pre-stimulus EDL immediately before the tactile stimulus at the perceptual threshold. Bars represent the mean and error bars represent the standard error of mean. Asterisks indicate significant simple main effects (*p* < 0.05). EDL, electrodermal level; EO, the eyes open; EC, the eyes closed.

Pre-stimulus EDL immediately before the stimulus whose intensity is at the perceptual threshold is shown in Figure 5. There was no significant main effect of controller (F (1, 12) = 3.065, *p* = 0.106, η^2^p = 0.203) or vision (F (1, 12) = 0.737, *p* = 0.407, η^2^p = 0.058). There was a significant interaction between the main effects (F (1, 12) = 5.688, *p* = 0.034, η^2^p = 0.322). Test of simple main effect revealed that the pre-stimulus EDL at the perceptual threshold in the external trials was significantly higher than that in the self-trials when the participant kept the eyes closed (F (1, 24) = 8.607, *p* < 0.05). Another test of simple main effect revealed that the pre-stimulus EDL at the perceptual threshold with the eyes closed was significantly higher than that with the eyes open in the external trials [F (1, 24) = 5.109, *p* = 0.033].

## 4. Discussion

### 4.1. Tactile Sensitivity

Tactile sensitivity was attenuated when the stimulus was self-triggered [15,16,17]. In those previous studies, the motor command was generated when the tactile stimulus was self-provided by active movement, but was not when it was provided by someone else. Efference copy of the motor command enables humans to predict the somatosensation caused by the motor command execution [18,19,20,21,22]. From this perspective, the efference copy of the motor command allows the participants to predict the tactile stimulus when they actively touch their body [2,14,17,23,24,25,26]. Thus, we hypothesized that the prediction of the stimulus via efference copy of the motor command is the cause of the tactile attenuation when the stimulus intensity is self-controlled (Hypothesis 1).

In the present study, the stimulus intensity at the perceptual threshold was significantly reduced when the participant controlled the stimulus intensity compared to when others controlled it. This result indicates that tactile sensitivity increases when the stimulus intensity is self-controlled. The η^2^p for this significant effect was 0.303. Accordingly, the effect size of the controller is considered to be large.

The motor command was produced in both the self-controlled and externally controlled trials. Therefore, in the present study, efference copy of the motor command is not the cause of the increase in tactile sensitivity during the self-controlled tactile stimulus. The present finding did not reject Hypothesis 1 that the attenuation of tactile sensitivity caused by self-triggered stimuli is due to the stimulus prediction via efference copy of the motor command.

The stimulus intensity at the perceptual threshold was lower when the participants could predict the timing of the stimulus [27]. Based on this previous finding, we formulated Hypothesis 2 that time prediction of the tactile stimulus alters the tactile sensitivity. However, our finding did not support this hypothesis. The participants were familiar with the dial number near the perceptual threshold due to the preliminary practice conducted with the eyes open. This indicates that the participant could predict the timing of the stimulus by viewing this number of the actual dial. This finding was contrary to a previous finding showing reduced ticklishness when a participant viewed the hand of someone else that tickled the body of the participant [3]. The participant could view the actual dial only when they turned it themselves. Nevertheless, no significant interaction was found between the controller and vision on the stimulus intensity at the perceptual threshold. Those suggest that time prediction via visual feedback from the number of the dial indicating stimulus intensity did not make a difference in tactile sensitivity between self-controlled and externally controlled stimuli. Thus, greater perceptual sensitivity to the self-controlled tactile stimuli is unlikely to be explained by time prediction.

In the present study, an actual dial controlling the stimulus intensity was turned either by the participant or someone else. The velocity of turning the dial was not the same between those variables. When the eyes were open, the dial number at the perceptual threshold was lower in the dial held by the participant. However, when the eyes were closed, that was higher in the dial held by the participant. This suggests that the velocity of turning the dial by the participant depended on whether their eyes were open. Lamontagne et al. (2007) demonstrated that individuals with stroke can adjust their walking speed by changing the speed of optic flow, suggesting that movement velocity is dependent on visual information [40]. According to this previous finding, the present finding may also reflect that vision influences movement velocity. This finding contradicts another finding showing that the stimulus intensity at the perceptual threshold was constantly lower when the dial was controlled by the participant, regardless of vision. This inconsistency suggests that the velocity of turning the dial does not account for the difference in tactile sensitivity between self-controlled and externally controlled tactile stimuli.

As demonstrated, neither the prediction of the stimulus nor the velocity of turning the dial explains the difference in tactile sensitivity between self-controlled and externally controlled tactile stimuli. The difference in the dial-tuning velocity between the self-controlled and externally controlled tasks depended on vision. Nevertheless, the stimulus intensity at the perceptual threshold was lower when the participant controlled the stimuli intensity regardless of vision. The participant knew whether the stimulus intensity was controlled by them or by someone else before each trial began, because an experimenter instructed it before each trial. Accordingly, only the difference in the condition between the self-controlled and externally controlled tactile stimuli is participant’s awareness of the self-control of the stimulus intensity. Therefore, our finding suggests that awareness of self-control over tactile stimulus intensity enhances tactile sensitivity.

### 4.2. Pre-Stimulus EDL

In the present study, a significant interaction was observed between the main effect of controller and that of vision on the pre-stimulus EDL. This suggests that vision and awareness of the self-control of the stimulus intensity interactively influence pre-stimulus EDL immediately before the tactile stimulus at the perceptual threshold. The η^2^p of this significant interaction was 0.322, indicating a large effect of the interaction between vision and awareness of the self-control of the stimulus intensity. A test of simple main effect revealed that pre-stimulus EDL at the perceptual threshold of the self-controlled tactile stimuli was significantly lower than that at the perceptual threshold of the externally controlled tactile stimuli, especially in the eye-closure condition. Furthermore, pre-stimulus EDL in the eyes-open condition was significantly lower than that in the eyes-closed condition particularly for the externally controlled stimuli. Taken together, pre-stimulus EDL at the perceptual threshold was the highest for the externally controlled tactile stimuli with the eyes closed.

Electrodermal activity is commonly measured as an indicator of physiological arousal [41]. Electrodermal response to a startle probe varies according to arousal levels [42]. Electrodermal responses triggered by threats were positively correlated with amygdala activation [43]. The amygdala is the area that plays a key role in processing fear responses [44]. More importantly, cognitive stress has been shown to increase EDL [32]. Based on this, the present finding suggests that cognitive stress increases when participants are aware that someone else is controlling the stimulus intensity and when visual information about the stimulus is unavailable.

On the one hand, when the participant controls the stimulus intensity themselves, they can predict the timing of the initial tactile perception through efference copy of the motor command for dial turning. On the other hand, the participant is aware that they cannot predict it via efference copy when stimulus intensity is externally controlled. When the eyes are closed, the participant cannot predict the timing of the initial tactile perception by viewing the number of the dial. Stress alters the process of sensation [45]. Thus, the present finding suggests that cognitive stress increases when the timing of the initial tactile perception is difficult to predict. Taken together, Hypothesis 3, the effect of the self-generated tactile stimulus on sympathetic activity is due to the prediction of the tactile stimulus without the influence of efference copy, was not supported by our present finding.

### 4.3. Limitation

The sample size was as small as 13 in the present study. This number of the participants was determined via a previous study that found a significant difference in tactile perceptual threshold between the conditions [34]. Despite this procedure, this small sample size potentially increases a risk of type II error.

## 5. Conclusions

The stimulus intensity at the perceptual threshold, when controlled by the participant, was significantly lower than when controlled by someone else, regardless of vision. This suggests that awareness of the self-control of the tactile stimulus intensity enhances tactile sensitivity. With the eyes closed, electrodermal levels immediately before the perceptual threshold stimulus were significantly lower when participants controlled the stimulus intensity compared to when another person controlled it. Furthermore, electrodermal levels immediately before the perceptual threshold stimulus, when triggered by another person, were significantly higher with the eyes closed. These findings suggest that cognitive stress increases when the timing of the initial tactile perception is difficult to predict. The present findings may contribute to understanding of the patients with tactile perception disorders. In future studies, further insight into the mechanism underlying the change in the tactile sensitivity caused by the awareness of the self-controlled stimulus intensity is needed.

## Data Availability

The data presented in this study are available on request from the corresponding author due to privacy restrictions.
